# The impact of environmental conditions on lifestyle quality in industrial and non-industrial region in the Czech Republic

**DOI:** 10.3389/fpubh.2025.1505170

**Published:** 2025-04-02

**Authors:** Petra Riedlova, Hana Tomaskova, Hana Slachtova, Jana Babjakova, Vitezslav Jirik

**Affiliations:** ^1^Centre for Epidemiological Research, Faculty of Medicine, University of Ostrava, Ostrava, Czechia; ^2^Department of Epidemiology and Public Health, Faculty of Medicine, University of Ostrava, Ostrava, Czechia; ^3^Institute of Hygiene, Faculty of Medicine, Comenius, University in Bratislava, Bratislava, Slovakia

**Keywords:** air pollution, lifestyle, industrial region, public health, environmental

## Abstract

**Background:**

Long-term exposure to air pollution is associated with a higher incidence of various non-infectious diseases. However, not only air pollution, but also other risk factors, such as lifestyle, can play a role in the occurrence of these diseases or premature deaths from them. The study aimed to compare the lifestyle of residents of two differently air polluted regions and to determine how lifestyle is affected by socioeconomic variables.

**Methods:**

In the framework of the project Healthy Aging in Industrial Environments, two cohorts of persons from an industrial area and a control area were established. The cohorts consisted of individuals aged 35 to 65 years. Lifestyle factors included diet, BMI, alcohol and cigarette consumption, duration of sleep, physical activity, and time spent doing hobbies. Influencing factors included region, sex, age, education, family status, and economic situation. Fully adjusted binary and ordinal logistic regression models were used for evaluation, and the output was the odds ratio (OR) with 95% confidence intervals (CI).

**Results:**

The effect of more air polluted industrial region was related to higher BMI (OR = 1.23; 95% CI: 1.08–1.4) and physical activity (OR = 1.31; 95% CI: 1.13–1.51) and surprisingly to lower smoking level (OR = 0.84; 95% CI: 0.74–0.99).

**Conclusion:**

The results of our study are useful in targeting public health strategies and intervention programs to specific populations, and the results will be share with public awareness groups that focus on prevention and the physiological aspects of physical activity.

## Introduction

### Effects of air pollution

Long-term exposure to air pollution has been associated with a higher incidence of various non-communicable diseases (NCDs) (1–3). Not only air pollution, but also other risk factors such as lifestyle can play a role in the incidence, morbidity, and mortality of particular diseases (1, 4). Air pollution can have a significant impact on lifestyle factors (diet, alcohol, smoking, body mass index, sleep duration, and physical activity) and exacerbate their negative health effects. Research shows that air pollution can increase oxidative stress and inflammatory processes in the body, which further amplifies the harmful effects of smoking and unhealthy diets. Smoking combined with air pollution significantly increases the risk of respiratory and cardiovascular disease. An unhealthy diet and low physical activity, together with air pollution, can increase in the chance of developing, obesity, insulin resistance, and metabolic disorders (5). The sedentary lifestyle contributes to a higher prevalence of chronic non-communicable diseases such as diabetes and hypertension (6), but overall, lifestyle risk factors, together with air pollution, also have a significant impact on the cognitive function of humans (1, 7, 8). On the other hand, a healthy lifestyle promotes a lower risk of oncologic disease, as well as various other NCDs (9, 10). Commonly modifiable risk lifestyle factors (presence of smoking, excessive alcohol consumption, unhealthy diet and physical inactivity) have been integrated into policies, strategies, and action plans in most developed countries (11), and modifying them, such as aforementioned regular physical activity, reduces the risk of morbidity but also mortality in people living in environmentally polluted regions (12). The combination of unhealthy living conditions and prolonged exposure to air pollution poses a significant risk to public health and, therefore, it is important to take measures both to improve the living conditions of individuals and to reduce exposure to air pollution (13).

### Study objectives

The study aimed to compare the lifestyle of residents of two different environmentally burdened locations in the Czech Republic and to determine how lifestyle (diet, alcohol, smoking, sleep, leisure time and physical activity) is affected by socioeconomic variables (sex, age, region, education, family and economic status).

## Methods

### Description of study participants and study design

The study included 3,528 volunteers from two regions that are environmentally burdened differently, i.e., industrial (IA) and nonindustrial (NA) areas in the Czech Republic. Volunteers within the project ´Healthy Aging in Industrial Environments´ (HAIE) completed a socioeconomic questionnaire (SES) after signing an informed consent, which was approved by the Ethics Committee of the University of Ostrava (No. 2/2018). The questionnaire included basic socioeconomic data on SEX, AGE, REGION, EDUCATION (EDU), FAMILY, and ECONOM, in addition to the questions about the lifestyle. This group included questions about food consumption and diet (DIETgrp), subjective assessment of diet (DIETsubj), some anthropometric factors (BMIgrp), alcohol consumption (ALCfreq, ALCfreq_risk), smoking status (SMKgrp), length of sleep (SLEEP), leisure time (LEISUREgrp), and physical activity (LTEQgrp- Leisure Time Exercise Questionnaire) (14). The description and content of each of the variables measured and the criteria for inclusion of probands in the study are presented in Table 1.

**Table 1:** Description of individual variables and inclusion criteria.

Variables	Description of variables	Evaluation criteria
SEX	M = male, F = female	x
AGEgrp	category 35–40, 41–45, 46–50, 51–55, 56–60, 61–65 years	x
REGION	NA = non-industrial area, IA = industrial area	NA = 5-year average annual concentration of PM_2.5_ was less than 12 μg/m^3^, 5-year average annual concentration of PM_10_ was up to 20 μg/m^3^ IA = 5-year average annual concentration of PM_2.5_ was more than 20 μg/m^3^, 5-year average annual concentration of PM_10_ was up to 30 μg/m^3^ residence for at least half of life and at least the last 10 years in one of the two regions of the Czech Republic (IA or NA), including at least 5 years in childhood (before the age of 15) (33, 34)
EDU	1 = elementary school and vocational school, 2 = high school with a graduation degree, 3 = higher extension schools and universities	x
FAMILY	0 = lives with someone, 1 = lives alone (risk)	x
ECONOM	0 = economically inactive, 1 = economically active (risk)	x
DIETgrp	0 = healthy food, 1 = unhealthy food	0 = 1–9 balanced diet 1 = 10–12 unbalanced diet (35)
DIETsubj	0 = satisfied with the diet, 1 = dissatisfied with the diet	x
BMIgrp	category 1 = min-24.9, 2 = 25–29.9, 3 = 30–34.9, 4 = 35–39.9, 5 = 40 kg/m^2^-max	x
ALCfreq	category 1–5	1 = frequent alcohol consumption 2 = 1-2x per week 3 = 1-3x per month 4 = <1x per month 5 = almost not at all
ALCfreq_risk	1 = poor consumption, 2 = medium consumption, 3 = strong consumption	Poor consumption = <1x per month, almost not at all Medium consumption = frequency 1-3x per month Strong consumption = 1-2x per week or more
SMKfreq	category 1–5	1 = non-smoker 2 = ex-smoker 3 = smoker - up to 5 cigarettes per day 4 = smoker - 6-19 cigarettes per day 5 = smoker - 20 or more cigarettes a day
SMKgrp	0 = non-smoker + ex-smoker, 1 = smoker	0 = non-smoker + ex-smoker 1 = smoker - up to 5 cigarettes per day +6–19 cigarettes per day +20 or more cigarettes a day
SLEEP	0 = 6–9 h of sleep, 1 = <6 a > 9 hod (risk)	x
LEISUREgrp	0 = 8-max, 1 = 0–7 (risk)	Number of hours /per week - free time for yourself
LTEQgrp	0= >24 = active 1 = 14–23 = moderately active and < 14 = insufficiently active/sedentary	Average weekly amount of physical activity during the past month at four levels of intensity (strenuous, moderate, moderate physical activity and sitting). The LTEQ questionnaire ascertains the physical activity that the respondents engaged in during their free time. In the LTEQ assessment, the weekly frequency of strenuous, moderate or moderate physical activity is multiplied by the numbers 9, 5 and 3, and the final score is calculated as the sum of these components (36)

This is a cross-sectional study that was conducted by randomly selecting a population approximately equally distributed between the two regions, but also with an equal distribution of age and gender.

Alcohol consumption as a risk factor was divided into 5 categories in the first stage, but the alcohol risk factor (ALCfreq_risk) already entered the fully adjusted model, combined into three categories. Smoking was also treated similarly. In the first stage, it was divided into 5 categories, with SMKgrp already entering the fully adjusted model in only two categories. More details are presented in Table 1.

### Statistical analysis

Basic descriptive statistics (mean, standard deviation, frequency tables) were used to describe the population. Basic comparisons between regions were made using the chi-square test for two samples. Binomial logistic regression models (DIETgrp, DIETsubj, SMKgrp, SLEEP, LEISUREgrp and LTEQgrp) and ordinal regression (BMIgrp and ALCfreq_risk) were used to analyze the effect of sociodemographic factors on lifestyle. First, a crude model was calculated that does not take into account any adjustments for possible confounding variables. Results of the fully adjusted model were expressed as OR (Odds Ratio) with 95% confidence intervals (CI). Statistical tests were assessed at the 5% level. Stata version 17 software was used for evaluation.

## Results

The study samples consisted of 3,528 volunteers from two environmentally distinct areas. The results of the descriptive statistics show that a higher number of persons came from the industrial area (IA) (64%), while the remaining number of volunteers (36%) came from the non-industrial area (NA). The average age of respondents entering the study was 49.8 ± 8.32 years in IA and 48.1 ± 7.08 years in NA. A statistically significant difference was found between the age groups in the regions and was also it was detected within BMI groups. In IA, the largest representation was group 25–29.9 kg/m^2^, while in NA, the largest representation was group min-24.9 kg/m^2^. In general, there is a higher percentage of participants in the lower BMI categories 1 and 2 in NA, coparing to a higher percentage of participants in BMI categories 3, 4, and 5 in IA. In the variable DIETgrp, the majority of respondents (in both regions) were found to have a healthy diet (88.4% in IA and 87.1% in NA). This was also associated with greater subjective satisfaction with the diet (56.3% in IA and 58.1% in NA), compared to those who were unhappy. In terms of education, the highest percentage of people in both areas was in category 2, i.e., high school with a graduation degree. The family status (FAMILY) of the people who entered the study was also similar in both regions. A higher number of people lived in a household with someone else (72.1% in IA and 73.3% in NA). More people were economically active in NA (90.1%). The proportion of the economically active was 84.0% in IA. Next, leisure time (LEISUREgrp), i.e., the number of hours a person has for himself/herself, was assessed. A statistically significant difference was found in this variable. 63.7% of people in IA had more than 8 h of free time per week, while in NA it was 60.3%. A statistically significant difference between the regions was also determined in physical activity (LTEQgrp). IA had a lower percentage of people (44.1%) who were physically active than NA (51.3%). For the SLEEP variable, the vast majority of respondents in both regions reported sleeping between 6 and 8 h per day (91.4% in IA and 93.2% in NA). For the risk factor ALCfreq_risk, no statistically significant differences were found between regions, but the results show the lowest alcohol consumption, but also the lowest number of people in category 5 (I almost do not drink alcohol at all). The last factor evaluated was SMKgrp. A significantly higher percentage of smokers was found in NA than in IA. Demonstrations of the distribution of variables and individual risk factors in the industrial and non-industrial region and results of statistically significant differences between regions, are presented in detail in Table 2.

**Table 2:** Description of the samples and demonstration of variables and risk factors in nonindustrial (NA) /industrial (IA) areas.

Variables	Category	Region IA (number) %	Region NA (number) %	*p*-value*
REGION		2,258 (64.0%)	1,270 (36.0%)	
SEX	M: male	986 (43.6%)	430 (33.9%)	<0.001
	F: female	1,272 (56.4%)	840 (66.1%)	
AGEgrp (years)	1: 35–39	261 (11.6%)	184 (14.5%)	<0.001
	2: 40–44	444 (19.7%)	278 (21.9%)	
	3: 45–49	462 (20.5%)	290 (22.8%)	
	4: 50–54	350 (15.0%)	242 (19.1%)	
	5: 55–59	378 (16.7%)	154 (12.1%)	
	6: 60–65	363 (16.1%)	122 (9.6%)	
BMIgrp (kg/m^2^)	1: ≤ 24.9	710 (31.6%)	498 (39.6%)	<0.001
	2: 25–29.9	906 (40.4%)	472 (37.5%)	
	3: 30–34.9	450 (20.1%)	213 (16.9%)	
	4: 35–39.9	140 (6.2%)	56 (4.5%)	
	5: ≥ 40	38 (1.7%)	20 (1.6%)	
DIETgrp	0: healthy food	1966 (88.4%)	1,091 (87.2%)	0.234
	1: unhealthy food	257 (11.6%)	162 (12.9%)	
DIETsubj	0: satisfied with the diet	1,130 (56.3%)	660 (58.1%)	0.329
	1: dissatisfied with the diet	877 (43.7%)	476 (41.9%)	
EDU	1: elementary school and vocational school	633 (28.1%)	341 (26.9%)	0.637
	2: high school with a graduation degree	915 (40.6%)	534 (42.1%)	
	3: higher extension schools and universities	708 (31.4%)	394 (31.1%)	
FAMILY	0: lives with someone	1,629 (72.1%)	931 (73.3%)	0.457
	1: lives alone	629 (27.9%)	339 (26.7%)	
ECONOM	0: economically inactive	361 (16.0%)	126 (9.9%)	<0.001
	1: economically active	1895 (84.0%)	1,142 (90.1%)	
LEISUREgrp	0: 8-max/h/per week	1,415 (63.7%)	753 (60.3%)	0.047
	1: 0–7 h/per week	805 (36.3%)	495 (39.7%)	
LTEQgrp	0: >24- active	995 (44.1%)	651 (51.3%)	<0.001
	1: 14–23- moderately active and < 14- insufficiently active/sedentary	1,263 (55.9%)	619 (48.7%)	
ALCfreq	1: frequent alcohol consumption	557 (24.7%)	356 (28.2%)	0.123
	2: 1-2x per week	629 (27.9%)	314 (24.8%)	
	3: 1-3x per month	599 (26.6%)	325 (25.7%)	
	4: <1x per month	318 (14.1%)	178 (14.1%)	
	5: almost not at all	148 (6.6%)	91 (7.2%)	
ALCfreq_risk	1: poor consumption	466 (20.7%)	269 (21,3%)	0.821
	2: medium consumption	599 (26.6%)	325 (25.7%)	
	3: strong consumption	1,186 (52.7%)	670 (53.0%)	
SLEEP (hours)	0: 6–9	2016 (91.4%)	1,145 (93.2%)	0.065
	1: <6 a > 9	190 (8.6%)	84 (6.8%)	
SMKfreq	1: non-smoker	1,233 (54.6%)	684 (53.9%)	0.041
	2: ex-smoker	556 (24.6%)	286 (22.5%)	
	3: smoker - up to 5 cigarettes per day	162 (7.2%)	87 (6.9%)	
	4: smoker - 6-19 cigarettes per day	232 (10.3%)	153 (12.1%)	
	5: smoker - 20 or more cigarettes a day	75 (3.3%)	60 (4.7%)	
SMKgrp	0: non-smoker + ex-smoker	1789 (79.2%)	970 (76.4%)	0.049
	1: smoker	469 (20.8%)	300 (23.6%)	

*chi2-test.

Table 3 shows the results of comparisons of both regions and other selected variables to each lifestyle factor after fully adjustment model for sex, age, education, family, and economic status.

**Table 3:** Analysis of lifestyle factors in relation to environmental conditions and socioeconomic factors.

		DIETgrp OR (95% CI)	DIETsubj OR (95% CI)	BMIgrp OR (95% CI)	ALCfreq_risk OR (95% CI)	SMKgrp OR (95% CI)	SLEEP OR (95% CI)	LEISUREgrp OR (95% CI)	LTEQgrp OR (95% CI)
RISK_region	NA	1 ref.	1 ref.	1 ref.	1 ref.	1 ref.	1 ref.	1 ref.	1 ref.
	IA	0.82 (0.66–1.01)	1.07 (0.92–1.25)	**1.23 (1.08–1.40)** ^**^	0.94 (0.82–1.08)	**0.84 (0.71–0.99)** ^*^	1.27 (0.97–1.67)	0.99 (0.85–1.15)	**1.31 (1.13–1.51)** ^***^
SEX	M	1 ref.	1 ref.	1 ref.	1 ref.	1 ref.	1 ref.	1 ref.	1 ref.
	F	**0.34 (0.28–0.43)** ^***^	**0.61 (0.52–0.71)** ^***^	**0.47 (0.42–0.54)** ^***^	**0.39 (0.34–0.45)** ^***^	**0.75 (0.63–0.89)** ^**^	1.03 (0.79–1.33)	**1.52 (1.31–1.77)** ^***^	**1.21 (1.05–1.40)** ^**^
AGE (years)	35–39	1 ref.	1 ref.	1 ref.	1 ref.	1 ref.	1 ref.	1 ref.	1 ref.
	40–44	0.78 (0.55–1.08)	0.82 (0.64–1.06)	1.10 (0.88–1.37)	0.83 (0.66–1.05)	0.86 (0.66–1.13)	1.04 (0.65–1.66)	0.76 (0.60–0.97)^*^	0.83 (0.65–1.06)
	45–49	0.66 (0.47–0.93)	**0.63 (0.49–0.82)** ^**^	1.19 (0.95–1.48)	0.82 (0.65–1.03)	**0.50 (0.38–0.67)** ^***^	1.34 (0.86–2.10)	**0.56 (0.44–0.71)** ^***^	0.93 (0.73–1.18)
	50–54	**0.60 (0.42–0.86)** ^*^	**0.73 (0.55–0.95)** ^*^	**1.47 (1.17–1.85)** ^**^	0.97 (0.76–1.24)	**0.53 (0.4–0.72)** ^***^	1.13 (0.70–1.83)	**0.42 (0.32–0.54)** ^***^	1.13 (0.88–1.45)
	55–59	**0.48 (0.33–0.71)** ^*^	**0.52 (0.39–0.69)** ^***^	**1.39 (1.09–1.75)** ^**^	1.07 (0.83–1.38)	**0.49 (0.36–0.67)** ^***^	1.00 (0.61–1.64)	**0.27 (0.21–0.36)** ^***^	1.23 (0.95–1.59)
	60+	**0.30 (0.19–0.47)** ^***^	**0.56 (0.41–0.75)** ^***^	**1.68 (1.31–2.15)** ^***^	0.97 (0.75–1.26)	**0.41 (0.29–0.57)** ^***^	0.86 (0.51–1.43)	**0.20 (0.15–0.28)** ^***^	**1.59 (1.21–2.10)** ^***^
EDU	1	1 ref.	1 ref.	1 ref.	1 ref.	1 ref.	1 ref.	1 ref.	1 ref.
	2	**0.72 (0.56–0.92)** ^***^	**0.59 (0.49–0.71)** ^***^	**0.75 (0.64–0.88)** ^***^	**1.47 (1.25–1.72)** ^***^	**0.56 (0.46–0.67)** ^***^	**0.68 (0.51–0.91)** ^*^	0.90 (0.75–1.08)	**0.53 (0.45–0.63)** ^***^
	3	**0.62 (0.47–0.82)** ^***^	**0.36 (0.30–0.44)** ^***^	0.58 (0.49–0.68)	**1.62 (1.36–1.92)** ^***^	**0.28 (0.22–0.36)** ^***^	**0.54 (0.38–0.75)** ^***^	1.06 (0.88–1.28)	**0.43 (0.36–0.52)** ^***^
FAMILY	0 = lives with someone	1 ref.	1 ref.	1 ref.	1 ref.	1 ref.	1 ref.	1 ref.	1 ref.
	1 = lives alone (risk)	1.00 (0.79–1.27)	0.86 (0.73–1.02)	0.95 (0.83–1.09)	**0.7 (0.61–0.81)** ^***^	**1.43 (1.20–1.71)** ^***^	1.42 (1.09–1.86)	**0.67 (0.56–0.79)** ^***^	1.04 (0.89–1.21)
ECONOM	0-inactive	1 ref.	1 ref.	1 ref.	1 ref.	1 ref.	1 ref.	1 ref.	1 ref.
	1-active	0.61 (0.44–0.85)	0.90 (0.71–1.14)	0.85 (0.70–1.04)	**1.57 (1.28–1.93)** ^***^	0.87 (0.67–1.12)	0.6 (0.42–0.86)	0.95 (0.75–1.21)	0.94 (0.76–1.18)

+adjusted model.

Ref., reference category; OR, odds ratio; *(*p* ≤ 0.05) statistically small significant difference, **(*p* ≤ 0.01) statistically significant difference, ***(*p* ≤ 0.001) statistically large significant difference, NA, nonindustrial area; IA, industrial area; CI, confidence interval; EDU: 1–1: elementary school and vocational school; 2: high school with a graduation degree; 3: higher extension schools and universities. Bold is for results that are statistically significant.

Statistically significant differences between regions were found only for BMIgrp, SMKgrp and LTEQgrp. After comparing lifestyle risk factors differences between the regions, the relationships with other variables (SEX, AGE, EDU, FAMILY, ECONOM) were evaluated. The first factor evaluated was DIETgrp. No statistically significant difference was detected by sex, with better eating habits found in women. The risk decreased with age for both sexes. In the education variable assessed, the risk was highest in the first group (that is, those with primary education). The risk was lower among the economically active. The subjective evaluation of diet (DIETsubj) was also analyzed. For this variable, a statistically significant difference was found between the sexes, and for women, as for DIETgrp, it was a protective factor. The risk decreased also with age. Education affected subjective dietary evaluation; with those with primary education being the most at risk. Neither family nor economic status influenced DIETsubj.

When BMIgrp was assessed, a statistically significant relationship was determined between sex and education. Higher BMI values were found in women and it was also a greater risk factor. There was an increase in risk with age and, conversely, higher education decreased the risk of overweight and obesity. For alcohol consumption, statistically significant differences were detected between sexes; age did not play a role in our study, but there was a decrease in both regions. A statistically significant relationship was also found between lifestyle factors and education. For the assessed risk factor for smoking (SMKgrp), both gender and age played a role, there was a reduction in risk with increasing age. The level of education had a protective effect on this risk factor. People who lived alone were at increased risk. In the case sleep as a behavior factor (SLEEP), a statistically significant relationship was determined only between education and sleep. People with primary education and living alone were most at risk. Conversely, the economically active person had a decreasing risk of sleep deprivation. Another variable assessed was LEISUREgrp (leisure time for him/herself). A correlation was found between sex; women were at greater risk;the risk decreased with age. One of the protective factors was the family status, namely, when the person lived alone. The last variable was physical activity (LTEQgrp). Here, a statistically significant intersexual difference was detected; women were at greater risk than men. A difference by age was also found. Finally, it was also confirme that the higher the education, the higher the frequency of exercise, and therefore the risk is lower for those with higher education (more details in Table 3).

## Discussion

The main objective and the most important results were to compare lifestyles between two different regions (non-industrial area vs. industrial area). Before model adjustment, statistically significant differences in lifestyle were found between regions by age (AGE), BMI (BMIgrp), economic status (ECONOM), leisure (LEISUREgrp), physical activity (LTEQgrp) and smoking (SMKfreq, SMKgrp). After full model adjustment, statistically significant differences between regions were detected only in BMI (BMIgrp), smoking (SMKgrp) and physical activity (LTEQgrp). In the other variables, the differences were not statistically significant. Higher risks in IA, although statistically insignificant, were found for subjective assessment of diet (DIETsubj) and sleep (SLEEP). In the other variables (DIETgrp, ALCfreq_risk, LEISUREgrp), the OR was higher in NA. In opposition to our results, a study by Strak et al. shows that for most lifestyle risk factors, an unhealthy lifestyle is associated with higher exposure to air pollution, although in some cases these associations are weak (1). However, the different results may be because the study included a much larger number of respondents (387,152) aged from 19 years (our study included respondents with a minimum age of 35 years) and also included people over 65 years of age (our study had a cutoff age of 65 years).

Higher BMI values and thus higher risk were observed in IA. An et al. summarizing 66 studies reported that 44% positively associated air pollution and BMI, 44% reported null findings, and the remaining 12% found that air pollution had a negative impact on body weight (15). Another study also reported that air pollution has an adverse effect on body weight. It is associated with higher BMI, but also with overweight and obesity (16). This may be due to the fact that a person exercises less when there is unfavorable air. Overall, air pollution is a potential risk factor for weight status in adults, according to a study by Huang et al. (16). In contrast, another study by Strak et al. reported that overweight was associated with lower concentrations of air pollution and underweight with higher concentrations of air pollution (1). However, these results may differ from our study due to the fact that the study by Strak et al. included a higher number of respondents over 65 years of age (42.7%), who are therefore assumed to have lower physical activity and have a higher BMI independent of air pollution levels.

BMI is also related to the LTEQgrp mentioned earlier. Most studies found that higher levels of air pollution are negatively associated with physical activity. They point out that people living in areas with high levels of air pollution tend to have lower levels of physical activity, which may be due to reduced motivation to participate in outdoor activities due to poor air quality (15, 17), Also, a study by Ruopeng et al. reported that improved air quality was associated with an increase in physical activity (18). Studies also often look at information on whether regular physical activity is a protective factor in affected areas. This was also addressed in the review, which included 13 studies (19). Eight studies from different countries concluded that the risks of exposure to air pollution outweighed the benefits of regular physical activity. A further five studies concluded that regular physical activity may be protective against the negative effects of long-term air pollution, particularly in healthy adults (19). However, in our case, after adjustment, the physical activity in IA was higher than in NA. This refutes everything that has been discussed so far about physical activity, air pollution, age categories, and the associated BMI. This fact may be the result of educational (health promotion) activities in the industrial region. Residents of the polluted area are constantly under pressure knowing that they live in a polluted area and on this occasion, there is more awareness in physical activities.

Also, in another variable- SMKgrp, a statistically significant difference between regions was determined. The number of smokers was higher in NA, therefore the risk is higher in this region. The same result was also found in the study by Strak et al. However, the study also looked at ex-smokers as a separate group, with more ex-smokers living in nonindustrial areas (1). Our study, however, combines ex-smokers and non-smokers into one group. However, this did not have an effect on the actual outcome of the relationship between smoking and living in a polluted/non-polluted area. There are probably more possibilities as to why there are more smokers in NA versus IA. One reason may be already traditional habits, which in some NA may be deeply rooted in local customs and traditions, thus leading to higher smoking rates. There may be more alternative entertainment and leisure activities available in IA, which ultimately reduces the need to smoke as a leisure activity. Finally, there is also greater availability of antismoking education and campaigns, which may reduce smoking rates in the area. The study by Cesaroni et al. that looked at the association between air pollution and smoking also found no statistically significant relationship (1).

After comparing the relationships between lifestyle risk factors between regions, the association with other variables (SEX, AGE, EDU, FAMILY, ECONOM) were evaluated. The first factor being evaluated was DIETgrp, which was divided into healthy and unhealthy. Better eating habits, although statistically insignificant, were detected in women, which is understandable, as women are more likely to be the ones who are more concerned about their health and healthy eating. Diet is therefore a greater risk factor for men. In the education variable assessed, the risk was highest in the group with basic education. The more educated people were, the risk decreased. This is also supported by the study by Roos et al. The eating habits of men and women with higher education were more in line with dietary guidelines than those of men with primary education. The OR of adherence to dietary guidelines increased by 60–85% when moving from the primary to the higher education group (20). Furthermore, the diet was not influenced that by marital status even if one lived alone, which is in opposition to the study by Roos et al. which reported that the eating habits of married respondents were more in line with dietary guidelines (20). A statistically significant relationship was also found between economic status and die. This is an unsurprising result because it is assumed that if a person is unemployed, they cannot eat as healthily as an economically active person. This hypothesis is supported by a study by Roos et al., which says that an economically inactive person is more at risk than an active one. Adherence to dietary guidelines was lowest among the unemployed (20).

The subjective assessment of diet (DIETsubj) was also analyzed. Education also had an effect on subjective dietary evaluation, with risk decreasing with increasing levels of education. Neither family nor economic status had an effect on DIETsubj. Higher proportions of economically active persons were in NA (90.1%). These values may have an effect on the aforementioned satisfaction of people with their diet (DIETsubj). If a person is economically active, he/she can afford a better quality diet. Such a person is assumed to have a higher income than an inactive person. The level of education could also affect DIETsubj, but we did not confirm this hypothesis in the study.

When BMIgrp was assessed, a statistically significant relationship was found between sex and education. Higher education decreased the risk of overweight and obesity. This may be due to the education of people on higher weight and various diseases associated with it.

For alcohol, statistically significant differences, were determined between sexes, with women consuming less alcohol, as well as other studies reporting that a higher prevalence was reported for men. Similarly, daily use was significantly higher in men than in women (1, 15). Age did not play a role in alcohol consumption in our study, while it did in the study by Strak et al. or Mravcik et al. (1, 21). Consumption of excessive amounts of alcohol on a single occasion is more common in the younger group (15–34 years), while daily alcohol consumption increases with age and is highest among 55–64 years and also 65+ (21). In our study, on the other hand, there was a decrease related to age in both regions. A statistically significant relationship was also found between EDU and alcohol consumption. The higher the EDU, the higher the chance of risky drinking was detected. Conversely, a study by Droomers et al. reported that excessive alcohol consumption was more common in groups with less education (22). These different results may be due to factors such as stress in our study. It is known that people with higher education are more stressed at work and therefore may try to reduce and suppress stress by using alcohol. It is also interesting to note that economically active people were at greater risk. However, it is possible that they are economically active who have the finances to buy alcohol, perhaps explaining the higher consumption rates of the active compared to the unemployed. In contrast, those who live alone are better off and at less risk.

For the assessed risk factor for smoking (SMKgrp), both gender and age played a role. Male preponderance, and therefore higher risk, was found in both our study and the study by Spilkova et al. The most common age category was between 18 and 29 years (23). In our study, although the age category was shifted to 35 years, there was a reduction in risk with increasing age. The level of education also had a protective effect on this risk factor. The more educated the respondents were, the less they smoked and the lower the risk. Furthermore, in a study by Spilkova et al. it was found that people with higher education reported smoking significantly lower often than those with less education (23). People who lived alone were also at increased risk, but also in greater numbers in our case. This suggests that people do not have to deal with smoking in living areas, for example, because it does not bother anyone. The study by Spilkova et al. reported that divorced people smoked more than any other group. It was surprising not to find a relationship between economically active and inactive people. We would have assumed that people who are not economically active would not have enough money for cigarettes, but this was not proven. The same result is also reported in a study by Spilkova et al. (23).

The sleep factor (SLEEP) was also assessed, where a statistically significant relationship was found for education, family and economy status and sleep. In other studies, relationships were detected between sleep and sex. In general, stronger relationships were observed for women compared to men, and women themselves also reported significantly poorer sleep quality (24, 25). Regression analyses showed that women were almost twice as likely to have poor sleep quality than men (26). This is assumed because women, usually due to pregnancy, motherhood, and parenthood, do not have such deep and high-quality sleep. The fact that these relationships were not also confirmed in our study may be due to the higher age category of the women entering the study. In our case, the women were over 35 years old. In contrast, increasing age was associated with poorer sleep in men, but also with overall poor sleep quality in both sexes (26, 27). The length of sleep can depend on physical activity during the day. The more physical activity a person has, the more tired they are and the better they sleep. At the same time, levels of hormones such as endorphins increase, which contributes to a sense of well-being and reduced stress. Lower stress can lead to better sleep (28). Another hormone that increases during movement is serotonin; this is a precursor to melatonin (a hormone that regulates the sleep cycle). Therefore, higher levels of serotonin lead to better melatonin production (29). Regular activity also helps regulate levels of the hormone cortisol, which is associated with stress. Its lower levels may thus contribute to better sleep quality (30). Last but not least, physical activity done outdoors helps regulate circadian rhythms, which can also promote better sleep cycles and melatonin production (19). However, if people live in a polluted area, they may not be as physically active, and therefore they sleep less well. Zanobetti et al. state that air pollution can negatively affect sleep quality, which is another major risk factor for the development of various chronic diseases (31). This is also proven by studies by Liu et al. and Yu et al., showing that higher concentrations of air pollution were negatively associated with the duration of sleep among survey participants (25, 32). However, none of the hypotheses regarding sleep were confirmed in our study. Subsequently, it was found that there is a risk of poor sleep in people living alone. Which can be satisfied with life satisfaction and better sleep. Active people, on the other hand, had a lower risk of falling asleep. This may be due to less stress in the case of regular income of working people.

Another variable assessed was LEISUREgrp (leisure time for self). A correlation was found between sex, with females at greater risk, with risk decreasing with age. One of the protective factors was the family status, namely when the person lived alone. This is undoubtedly due to the fact, that these people have more time for themselves and their hobbies. Leisure time is often associated with physical activity.

Thus, the last variable was physical activity (LTEQgrp). The oldest age groups being the most at risk, which is understandable as the ability to engage in physical activity decreases with age. Finally, it was also found that the higher the education, the higher the frequency of exercise, and therefore the risk is lower for those with higher education. Physical activity and its association with BMI, leisure time, and associated air pollution have been discussed more fully above for other variables.

A limitation of the study considering potential inaccuracies include the subjectivity of the assessment in the writing of the respondents’ questionnaires, which probably may have been most evident in the results of the BMI values, where respondents may not have known their exact weight and height, self-reported values can be imprecise as well. The least reliable results could be the data on the frequency of alcohol consumption and smoking. These risk factors may have had the highest percentage of false responses. Respondents may underreport or overreport these behaviors due to social desirability bias or memory lapses. Another limitation may be the difference in the level of urbanization in both regions, which we did not deal with.

## Conclusion

In our study, the effect of environmental pollution was negative associated with BMI and physical activity and positive for smoking among 35-65-year-olds. Other factors, mainly sex, education, and age, are also involved in the lifestyle investigating factors. The female sex acts as a risk factor for leisure and physical activity. Higher education has a mostly positive effect on lowering alcohol consumption. Furthermore, older age is seems to be a protective factor beyond BMI and physical activity. Living without a partner is risky in association with smoking and is protective for alcohol consumption and leisure activities. Economically active people are at increased risk of alcohol consumption.

The results of our study are useful in targeting public health strategies and intervention programs to specific populations, and the results will be share with public awareness groups that focus on prevention and the physiological aspects of physical activity.

## Data Availability

The raw data supporting the conclusions of this article will be made available by the authors, without undue reservation.
